# From text to structured data: Converting a word-processed floristic checklist into Darwin Core Archive format

**DOI:** 10.3897/phytokeys.9.2770

**Published:** 2012-01-30

**Authors:** David Remsen, Sandra Knapp, Teodor Georgiev, Pavel Stoev, Lyubomir Penev

**Affiliations:** 1 Global Biodiversity Information Facility, Copenhagen, Denmark; 2 Department of Botany, Natural History Museum, Cromwell Road, London SW7 5BD, UK; 3 Pensoft Publishers, Sofia, Bulgaria; 4 National Museum of Natural History & Pensoft Publishers, Sofia, Bulgaria; 5 Institute of Biodiversity and Ecosystem Research & Pensoft Publishers, Sofia, Bulgaria

**Keywords:** Data mining, taxonomic checklists, Darwin Core Archive

## Abstract

The paper describes a pilot project to convert a conventional floristic checklist, written in a standard word processing program, into structured data in the Darwin Core Archive format. After peer-review and editorial acceptance, the final revised version of the checklist was converted into Darwin Core Archive by means of regular expressions and published thereafter in both human-readable form as traditional botanical publication and Darwin Core Archive data files. The data were published and indexed through the Global Biodiversity Information Facility (GBIF) Integrated Publishing Toolkit (IPT) and significant portions of the text of the paper were used to describe the metadata on IPT. After publication, the data will become available through the GBIF infrastructure and can be re-used on their own or collated with other data.

## Introduction

Data mining and converting texts to structured data, especially of historical biodiversity literature, is a major challenge in biodiversity informatics. Methods and tools developed to provide such conversions ideally should allow extraction of data from texts of both legacy and prospectively published literature and their incorporation into globally accessible databases (Kirkup et al. 2005; Brandenburger and Hagedorn 2006; Curry and Connor 2007, 2008; Agosti et al. 2007; Penev et al. 2011).

This paper describes a pilot project of such a conversion realized for the journal PhytoKeys. A conventionally written *Checklist of vascular plants of the Department of Ñeembucú, Paraguay* (De Egea et al. 2012), consisting of more than 4,100 taxon names, was submitted to PhytoKeys as a Microsoft Word file. After peer-review and editorial acceptance, the final revised version was converted into Darwin Core Archive format from the original manuscript and published both as a conventional paper in PhytoKeys and as DwC-A structured data through the Global Biodiversity Information Facility (GBIF) Integrated Publishing Toolkit (IPT). In addition and for convenience of the readers and data users, the same data are also published as a supplementary Excel file in an Appendix to the checklist (doi: 10.3897/phytokeys.9.2279.app1).

Checklists are often based upon personal or institutional databases of specimen records and names (see Acevedo and Strong 2012 and http://botany.si.edu/antilles/WestIndies/index.htm); part of the Ñeembucú checklist was assembled using a database, but the presentation in final form also involved editing and adding some data elements using a conventional word processing application. This combination of methods meant that recovering the entire dataset from the conventional MS Word file as described below was more efficient than combining the different elements derived from databases and word processed files *de novo*.

## The Darwin Core Archive Standard and Format

The Global Biodiversity Information Facility (GBIF) and the Biodiversity Information Standards (TDWG) recently launched a new format for storing species occurrence data and taxon checklists, named Darwin Core Archive (DwC-A) (http://www.gbif.org/informatics/standards-and-tools/publishing-data/data-standards/darwin-core-archives/). DwC-A is based on the Darwin Core (DwC) standard (Wieczorek et al. 2012) and seems to be a convenient and widely applicable format for handling primary biodiversity data, including conversions from text into data. DwC-A is an internationally accepted biodiversity informatics data standard and the preferred format for publishing data through the GBIF network.

Each Darwin Core Archive consists of at least three files:

1. One or more data files keeping all records of the particular dataset in a tabular format such as a comma-separated or tab-separated list;

2. The archive descriptor (meta.xml) file describing the individual data file columns used, as well as their mapping to DwC terms; and

3. A metadata file describing the entire dataset which GBIF recommends be based on EML (Ecological Metadata Language 2.1.1).

The format is defined in the Darwin Core Text Guidelines (http://rs.tdwg.org/dwc/2009-02-20/terms/guides/text/index.htm). The Darwin Core is no longer restricted to occurrence data only, and together with the more generic Dublin Core metadata standard (on which its principles are based), it is used by GBIF and others to encode metadata about organism names, taxonomies and species information.

GBIF has produced a series of documents and supporting tools that focus primarily on Darwin Core publishing. They are divided into three profiles, each of which represents a series of documents based on the different content types upon which GBIF focuses:

• Primary biodiversity data: http://www.gbif.org/informatics/primary-data/publishing/

• Checklists: http://www.gbif.org/informatics/name-services/publishing/

• Resource metadata: http://www.gbif.org/informatics/discoverymetadata/publishing/

In addition to the GBIF Integrated Publishing Toolkit (IPT), two additional tools have been developed for producing Darwin Core Archives:

1. A suite of MS Excel Templates (http://tools.gbif.org/spreadsheet-processor) that are coupled with a web service that processes completed files and returns a validated Darwin Core Archive. Templates exist for primary biodiversity data, simple checklists, and EML metadata.

2. Darwin Core Archive Assistant (http://tools.gbif.org/dwca-assistant) is a tool that composes an XML metafile, the only XML component of a Darwin Core Archive. It displays a drop-down list of Darwin Core and extension terms, accessed dynamically from the GBIF registry, and displays these to the user who describes the data files. This allows Darwin Core Archives to be created for sharing without the need to install any software.

Darwin Core Archive files can also be generated from data uploaded on the IPT and then published as a zipped supplementary file, associated with a research article, for example species occurrence data and checklists underlying any taxonomic revision (see sample papers by Talamas et al. 2011; Faulwetter et al. 2011; Lambkin and Barlett 2011). The publication of large datasets in the form of data papers is also supported (Chavan and Penev 2011, see Narwade et al. 2011 for an example of such a data paper).

## The conversion process

Checklists in botany traditionally contain a series of elements, including accepted name, synonyms, habit (life form), distribution and vouchers (and sometimes many more). Vouchers are usually specimens or literature records cited to confirm the presence of the taxon in the region for which the checklist is prepared. Although checklists are seen as relatively standardized as compared to monographic or revisionary works there are a number of conventions used in their presentation that our conversion process revealed as potential problems for mining such literature. Print-based publication has a tradition of saving space to lower costs. This space-saving gives rise to conventions such as “microcitation” of places of publication of scientific names using single page references only (see RDM Page’s iPhylo at http://iphylo.blogspot.com/2011/03/microcitations-linking-nomenclators-to.html) or the abbreviation of generic names in lists (see Peña-Chocarro et al. 2010). Deconstructing a text-based checklist with such minimalisations can cause contextual problems such as, for example, a synonym with an abbreviated generic name can lose its context in a parsed checklist. These sorts of problems will be encountered in any conversion of text to data set, and our experience here has highlighted some of the issues that need to be taken into account before a conversion is attempted.

As an annotated species checklist, taxa are presented sequentially with a hierarchical order implied by the sequence. The higher taxa are listed first. In this checklist the highest groups are only presented with informal names (e.g., “Ferns” “Lycopods”). Latin family names are listed as child taxa of the aforementioned informal names. Species and infraspecies, which form the primary focus of the list, are presented alphabetically in separate lines that follow the listing of the family in which they are contained.

**Figure 1.**
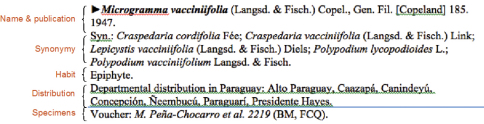
A typical species record from the checklist.

Figure 1 illustrates a typical species record from the Ñeembucú checklist with annotations on the left that identify major data sub-elements within the species account. It shows that a single taxon record occupies multiple lines in the manuscript. Different lines in a record hold different data subelements. The figure illustrates a complete record but the records in the manuscript are not completely standard and may not contain all the sub-elements as shown in the figure. For example, a species with no recognised synonyms would lack the synonymy section, or a taxon identified only to a genus level would lack publication information.

A complete record has the following parts.

The first line contains the taxon name and authorship followed by a short bibliographic citation representing the original publication of the name.

The second line contains a list of synonyms, with authorship, separated by semicolons.

The third line describes the habit of the referenced taxon with values obtained from a short set of possible values.

The fourth line details the distribution of the taxon in the rest of Paraguay by Department.

The fifth line refers to voucher material showing the presence of the taxon in the Department of Ñeembucú.

The objective in the following steps was to deconstruct these records from the unparsed manuscript, split them into smaller atomic parts, and reassemble these parts into a database structure that could then be mapped to the standard Darwin Core Archive format.

The tools used for this process were a text editor that supports regular expressions and a simple relational database “application” or “management system” (RDBMS). Regular expressions are coding conventions, similar to a programming language, used to recognise and manipulate patterns in text data (Friedel 1998). They were utilised to split the data into smaller parts for assembling into our database.

Following the analysis of the manuscript, we designed a target database structure to which the data were transformed. Our target format is based on the same structure as the Darwin Core Archive format for annotated species checklists. The checklist data would be parsed and transformed into three data tables ultimately stored as simple tab-delimited files with a structure as follows (see Excel data tables at: DOI: 10.3897/phytokeys.9.2279.app1).

This table forms the taxon record component (Table 1) of the DwC-A and is stored in a Darwin Core Archive core structure. Additional repeating data elements are stored in supplementary text files, called *extensions*. There are extension definitions for many data subtypes found in annotated species checklists such as distribution data, common names, and descriptive content. We designated two additional tables; one for distribution data (Table 2) and one for descriptive elements (Table 3). The first extension table stores the presence of the taxon in Paraguay and distribution in country's departaments.

**Table 1.** Taxon table - One row per name.

ID	A unique identifier for each name in the database including synonyms, higher taxa and the accepted name itself. This is the key element for the data interoperability. All related data refer to this identifier
Scientific Name	Stores the full (with authorship) scientific name of the taxon
Taxonomic Status	Identifies whether it is an accepted name or a synonym
Rank	Indicates the rank of the taxon
Accepted name ID	In case of synonym, this field indicates the ID of the accepted name
Parent ID	In case of accepted name, this field indicates the ID of its taxonomic parent
Nomen. Status	For synonyms containing also nomenclatural comments (e.g., “comb. Superfl.”)
Original Publication	Indicates the original publication where the taxon was published
Remarks	Stores additional remarks regarding the taxon

The second extension table stores the voucher information, as well as the taxon habit.

**Table 2.** Distribution table - One row per geographic region per taxon.

ID	The ID of the taxon being referenced
Country	Stores Paraguay as a constant value
Locality	Stores the department name(s)

The conversion of the *Checklist of vascular plants of the Department of Ñeembucú, Paraguay* was realized through the following procedures and steps:

**Table 3.** Description table - One row per description per taxon.

ID	The ID of the taxon being referenced
Type	The type of description being recorded (e.g., Habit, Genetic, Reproduction)
Description	The descriptive text itself

### Step 1 – Transform the checklist records into one taxon record per line

The checklist was cut from the original MS Word manuscript and pasted as text into the text editor. Two carriage returns separate the individual taxon records. Taxon records themselves occupy multiple lines with each line representing a major sub-element. Our goal in this step was to condense a multi-line taxon record into a single line and separate the record sub-elements with tabs. This is done in reverse order by:

replacing all double line endings with some unique character not in the manuscript (we used “@”);

replacing all remaining (single) line endings with a tab;

returning the original replacement in the first step (the “@”) with a line-ending.

The end result of this step is a formatted text that can now be imported into a relational database manager.

### Step 2 – Import the file into a relational database manager

**Figure 2.**
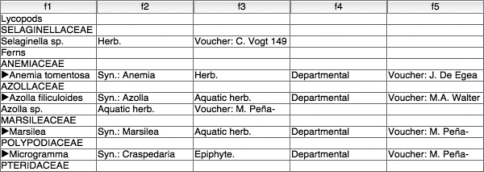
Taxon records imported into a database.

We imported the raw, re-formatted data into a relational database manager (Filemaker Pro). Figure 2 illustrates a portion of the result. A quick review reveals that the columns in individual records are not aligned. This is due to the fact that some species have incomplete set of subelements, as identified in Fig. 1. This issue is addressed in Step 4.

### Step 3 – Assign unique identifiers to each row

**Figure 3.**
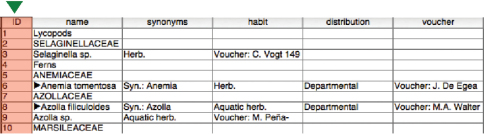
An updated database with final column titles and unique identifier added for each record.

We added a new column to the table and populated it with an incrementing integer to create a unique identifier for each record, as shown in Figure 3 (note that for convenience we named the individual columns with their target data type).

### Step 4 – Align content to correct columns

**Figure 4.**
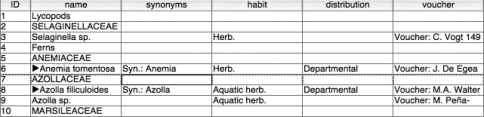
Data correctly aligned with columns.

This step requires the identification and transposition of data values from an incorrect column to the correct one. Identification of errant values was facilitated by the authors’ use of consistent and unique text in each subelement of a taxon record. For example, the synonymy section always starts with the text “Syn:” and the specimen data always starts with the text “Voucher.” The re-alignment process therefore consists of searching for instances of this standard text within other columns of the table, copying the resultant set of responses to the correct column, and deleting the original value from its erroneous location. At the end of this step, all data values were validated and placed in their correct columns, as shown in Figure 4.

### Step 5 – Build the taxonomic hierarchy

The Ñeembucú checklist is presented in a typical manuscript form where the hierarchical classification information can be inferred from the natural order of taxa presented in the list. Parent taxa are identified first, followed by child taxa. Knowledge of the relationships among taxon ranks and the means to identify members of a particular rank from the sequence presented is required to correctly infer the taxonomic structure.

We recorded taxonomic hierarchy information by adding a “parent ID” column to the core taxon table. This column will contain the identifier value for the immediate parent of the taxon record. For example, in Figure 4 we can infer that the parent taxon of *Azolla filiculoides* is AZOLLACEAE which has an ID of 7. The parent ID value for *Azolla filiculoides* is therefore 7. Likewise, the AZOLLACEAE record has a parent of “Ferns” and therefore it would have a parent ID of 4.

No automated techniques were used to populate this parent ID column but it was a straightforward exercise to copy and paste the correct values into the table. This was the only manual process in the entire transformation.

### Step 6 – Split the name from the original publication information

Some of the columns in the main data table require additional parsing. The first column contains the scientific name, original publication citation, and, in some cases additional notation indicating the taxon status.

**Figure 5.**
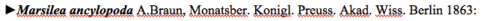
Example of a scientific name entry.

Figure 5 illustrates a typical example. For convenience in parsing, the authors separated the scientific name component of the line from the publication component using a comma (,). It was therefore straightforward to use a regular expression to split the content at the comma and separate the publication and name into separate columns. The ‘►’ character and other notations that occupy the first character of some records (see De Egea et al. 2012 for explanation of the meanings for these notations) were separated out to a distinct column in a similar way.

### Step 7 – Split the synonyms into separate records

**Figure 6.**
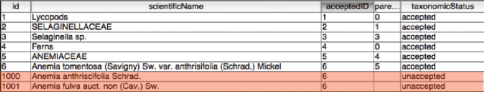
Synonym records (highlighted) added and linked.

Synonyms in the manuscript are separated by semicolons and the subsequent table stores all synonyms in a single field. Our objective in this step was to split each synonym into a separate name record in the main table and link it to the accepted name record via a new ‘accepted name ID’ field. We did this by exporting the ID and Synonym field from the table to a text file. Using a regular expression, we were able to split the synonyms into separate lines – one line per synonym and add the identifier to each line as well. This was then re-imported back into the database as new synonym records, linked to the accepted taxon via the ‘accepted name ID’. A ‘taxonomic status’ column was also added and populated with values. Incoming synonyms were labelled as ‘unaccepted’ names and accepted taxa were identified as ‘accepted’ (see Figure 6).

### Step 8 – Export distribution and voucher information as separate tables

In order to conform to the Darwin Core Archive standard, some of the data columns in the initial core table were moved to the separate Distribution and Description tables. These data were exported with the original unique ID to provide the relational link back to the core taxon record.

### Step 9 – Export the data to tab-separated text files

The final data processing step consisted of creating a set of three exported text files containing the core checklist data and the ancillary voucher and distribution data. This was achieved using the text export function of the database management system. Three text files were created as a result of this step.

### Step 10 – Map the exported text files to the Darwin Core Archive standard using the Darwin Core Archive Assistant

Each of the three exported data files was described using Darwin Core Archive Assistant. This web application is used to generate the XML ‘data map’ that specifies the Darwin Core standard term each column value is mapped to. It allows users of the data to understand, for example, that column 2 of the data file named “checklist.tab” is mapped to the Darwin Core term, *scientificName*.

### Step 11 – Use the Integrated Publishing Toolkit to generate a description of the dataset in the Ecological Metadata Format (EML)

The Darwin Core Archive format recommends the inclusion of a description of the dataset in the archive itself and specifies a metadata format for this. We used an installation of the Integrated Publishing Toolkit located at GBIF to compose the metadata document by filling in a series of web forms. The output of this exercise was the EML document. Note that in both steps 10 and 11 no underlying knowledge of XML is required to create these XML documents.

### Step 12 – Proof the final parsed output

The parsed checklist data was provided to the manuscript authors for proofing by exporting a copy of the data as an Excel spreadsheet file. Minor parsing errors were identified and corrected in the source database and the data export (Step 9) was repeated.

### Final step – Create the Archive

The final step in creating the Darwin Core Archive was to place all the generated files into a single folder and zip them into a single archive. This zip file is the Darwin Core Archive itself. It was validated using the Darwin Core Archive Validator (http://tools.gbif.org/dwca-validator/) and uploaded onto the Pensoft IPT Data Hosting Center (http://ipt.pensoft.net/ipt/resource.do?r=neembucu).

## Conclusions

This pilot project undertaken with the *Checklist of vascular plants of the Department of Ñeembucú, Paraguay* should be seen as a test of a necessary step in the process of creating a data conversion and publishing workflow for primary biodiversity data based on a new interoperability format, the Darwin Core Archive (DwC-A). Such a conversion workflow will allow essential taxonomic information, such as taxon checklists and catalogues to be published simultaneously in both human-readable text and machine-readable structured data formats.

Our example here shows that Darwin Core Archive format can be used for conversions of a text-based species occurrence checklists published in a traditional botanical format. The advantage of using Darwin Core Archive format is that the data structured according to DwC-A requirements can be published through the GBIF IPT, where the authors can also publish enriched metadata descriptions of the dataset. DwC-A structured data can easily be shared between scientists and publishers as a set of zipped files, that contain not only the data, but also the associated metadata (descriptions of the fields in the data tables and also description of the whole dataset) and the relations between them. Once the data are published through the GBIF infrastructure, they become discoverable through searches of metadata or through individual data items themselves (e.g., taxon names).

Such a conversion can be achieved when manuscripts are written following very strict text-formatting conventions and when various iterations of the decomposed text are proof-read by the original authors in order to catch any mistakes in parsing or resulting from parsing. While it might be preferable that such checklists are generated directly from a Darwin Core format database such that a conversion would not be necessary, in reality, checklists are produced in many formats and with many audiences in mind, not just with re-use of data as an ultimate objective. Very often scientists producing checklists are not necessarily in possession of sufficient database knowledge to design schemas that are really structured for re-use. We further face the reality that Darwin Core Archive - especially when used for sharing checklists - is a format new to most biologists and prior to our efforts here, no checklist conversion workflow has been available for the Darwin Core Archive format. Our experience in converting the *Checklist of vascular plants of the Department of Ñeembucú, Paraguay* (De Egea et al. 2012) will be useful for formulating text-formatting conventions, as well as for developing software tools that will facilitate conversion of text to data.

A couple of general principles for those creating checklists in text-based formats emerged:

1. Space saving conventions need to be abandoned or at least constrained.

2. Consistency in punctuation and in taxon entry format is essential; this would also apply to parsing of legacy data. A step in a conversion process that would reformat legacy text to a machine readable form will be time-consuming but ultimately might save re-inventing a process each time for differently formatted legacy texts.

In addition, the text decomposition and the problems we encountered while refining the conversion process will be useful when experimenting with the opposite process, the creation of human-readable text checklists from Darwin Core Archive data.

The simultaneous provision of data in both human and machine-readable form greatly improves their use and re-use. Text-based presentations will continue to be useful for checklists employed in on-the-ground conservation tasks such as protected area management. Transformation of a manuscript to a database greatly increases the potential for re-use as the data become available in a more atomized and standardized form. Examples of re-use of data published in this way include the re-aggregation of regional lists into larger compilations such as supra-departmental lists, national floras/faunas and global species catalogues. A converted checklist enables a regional list like this one to be integrated with additional species-level data such as that presented in the Encyclopedia of Life or GBIF and thus extend the annotations tied to the list. It allows also the data to be cross-referenced with additional taxonomic and nomenclatural indexing centres and registries such as the International Plant Name Index (IPNI), MycoBank, Index Fungorum or ZooBank where authorship, publication source and other taxonomic and nomenclatural components can be verified. Such a verified list can in turn serve as a taxonomic authority for new regional compilations.
